# Problems of primary T-cell lymphoma of the thyroid gland -A case report

**DOI:** 10.1186/1477-7819-10-58

**Published:** 2012-04-19

**Authors:** Junkichi Yokoyama, Shin Ito, Shinichi Ohba, Mitsuhisa Fujimaki, Eriko Sato, Norio Komatsu, Katsuhisa Ikeda, Makoto Hanaguri

**Affiliations:** 1 Department of Otolaryngology, Head and Neck Surgery, Juntendo University School of Medicine, Tokyo, Japan; 2 Department of Hematology, Juntendo University School of Medicine, Tokyo, Japan; 3 Department of Otolaryngology, Kyushu Rosai Hospital, Kitakyushu, Japan

**Keywords:** Peripheral T-cell lymphoma, Thyroid, Hashimoto’s thyroiditis, Molecular diagnosis, Gene rearrangement

## Abstract

In the following report we discuss a very rare case of malignant T-cell lymphoma of the thyroid gland that developed in a 70-year-old woman with a past history of hypothyroidism due to chronic thyroiditis. The chief complaint was a rapidly growing neck mass. CT and ultrasonographic examination revealed a diffuse large thyroid gland without a nodule extending up to 13 cm. Although presence of abnormal lymphoid cells in the peripheral blood was not found, the sIL-2 Receptor antibody and thyroglobulin measured as high as 970 U/ml and 600 ng/mL respectively. Fine needle aspiration cytology diagnosed chronic thyroiditis. A preoperative diagnosis of suspicious malignant lymphoma of the thyroid gland accompanied by Hashimoto’s thyroiditis was made, and a right hemithyroidectomy was performed to definite diagnosis. Histological examination revealed diffuse small lymphocytic infiltration in the thyroid gland associated with Hashimoto’s thyroiditis. Immunohistochemical examination showed that the small lymphocytes were positive for T-cell markers with CD3 and CD45RO. The pathological diagnosis was chronic thyroiditis with atypical lymphocytes infiltration. However, Southern blot analysis of tumor specimens revealed only a monoclonal T-cell receptor gene rearrangement. Finally, peripheral T cell lymphoma was diagnosed. Therefore, the left hemithyroidectomy was also performed one month later. No adjuvant therapy was performed due to the tumor stage and its subtype. The patient is well with no recurrence or metastasis 22 months after the surgical removal of the thyroid. As malignant T-cell lymphoma of the thyroid gland with Hashimoto’s thyroiditis was difficult to diagnose, gene rearrangement examination needed to be performed concurrently.

## Background

Malignant lymphoma of the thyroid gland is uncommon, representing only 2 to 5% of all thyroid malignancies, and is often associated with autoimmune disorders, such as Hashimoto’s thyroiditis [1]. Many reported cases are B-cell lymphomas of the thyroid, which include marginal zone B cell lymphoma of the mucosa-associated lymphoid tissue (MALT) type (maltoma) and diffuse large B-cell lymphoma. Primary T-cell lymphomas are extremely rare at less than 2% of all primary lymphomas of the thyroid gland. The present case report describes a rare case of primary T-cell lymphoma associated with Hashimoto’s thyroiditis that was difficult to diagnose pathologically. It was, therefore, useful to diagnose through means of genetic study.

## Case report

A 70-year-old woman was referred to our clinic, complaining of rapid anterior swelling at the neck. A diffuse firm goiter was observed and the patient indicated feeling oppression of the neck. No lymph node swelling was observed. The patient has a past history of hypothyroidism and enlargement 10 years earlier and takes 50 μg levothyroxinesodiumdaily. Laboratory examination indicated almost normal thyroid function (free T4 1.3 ng/dL, free T3 3.6 ng/dL, thyroid-stimulating hormone (TSH) 0.04 m unit/mL, thyroglobulin 600 ng/mL) with anti-microsome antibodies and anti-thyroglobulin antibodies. The s-IL-2 Receptor antibody measured as high as 970 U/ml.

**Figure 1:**
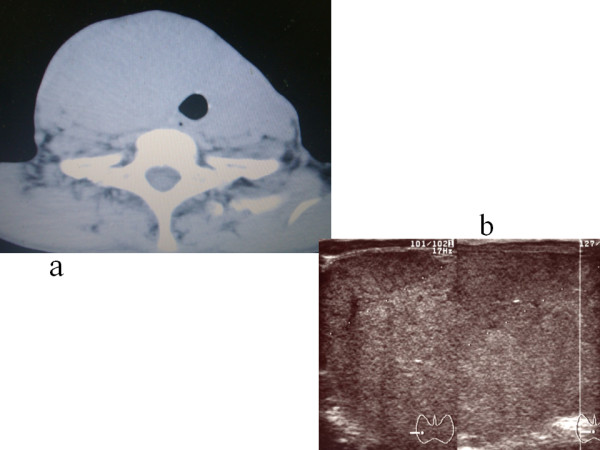
**CT and ultrasonographic examination.** (**a**) CT shows the diffuse enlarged thyroid gland without nodule. (**b**) Ultrasonographic examination demonstrates a homogenous enlargement of the thyroid gland not so low echogenecity as typical malignant lymphoma.

However, thyroxine-supplementation could not decrease the size of the thyroid. The thyroid gland continued to enlarge and diffuse, and was approximately 5 cm thick at the isthmus portion on the ultrasonographic examination. A computed tomography (CT) scan showed a huge thyroid enlargement with decreased internal density (Figure 1). There was no obvious mass lesion in the thyroid. The enlarged thyroid gland was well demarcated.

**Figure 2:**
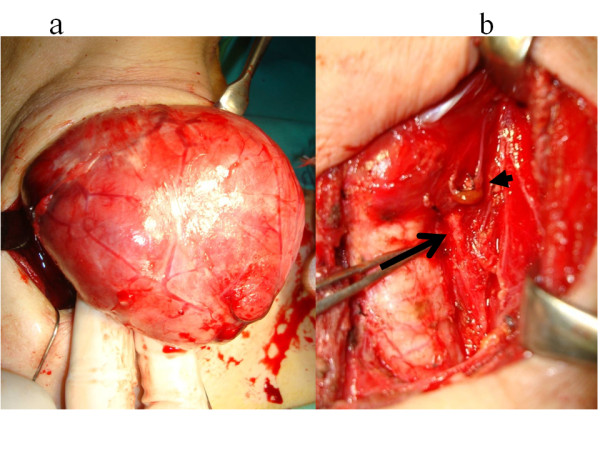
**Operative findings. a)** First operative finding: the right hemithyroidectomy was performed without any complication. **b)** Second operative finding: the arrow indicates a left recurrent laryngeal nerve. The arrow head indicates a parathyroid gland with supplying artery.

On FDG-PET (^18^ F-fluorodeoxy glucose-Positron Emission Tomography) examination, the FDG accumulation was detected in the enlarged thyroid region, suggestive of Hashimoto’s thyroiditis and/or lymphoma. Serological examinations were negative for human T-cell lymphoma virus type 1 (HTLV-1). Fine needle aspiration cytology suggested the case was consistent with Hashimoto’s thyroiditis. Although we suspected possible malignant lymphoma associated with Hashimoto’s thyroiditis from the large thyroid size and a rapid growth rate, the diagnosis was not confirmed. Therefore, the right hemithyroidectomy was performed to obtain a definite diagnosis and relief of the local symptoms caused by an enlarged goiter (Figure 2). Histological examination revealed diffuse small lymphocytic infiltration in the thyroid gland associated with Hashimoto’s thyroiditis. However, Southern blot analysis of tumor specimens revealed only a monoclonal T-cell receptor gene rearrangement. The final diagnosis was peripheral T cell lymphoma. Therefore, a left hemithyroidectomy was also performed one month later. No adjuvant therapy was performed because of the tumor stage and its subtype. The patient has been well with no recurrence or no metastasis. Furthermore, no additional medication has been required for 22 months since the surgery.

### Pathological findings

Macroscopically, the resected thyroid gland was diffusely enlarged, but the thyroid tumor was firmly encapsulated without extracapsular spread. The cut surface of the thyroid had pale white medullary components.

Histologically, severe and diffuse infiltration of the lymphoid cells was found in the medullary region. Lymph follicle formation with germinal centers was noted in the periphery of the medullary region. Atypical lymphoid infiltrations were also located among the atrophic thyroid follicles.

**Figure 3:**
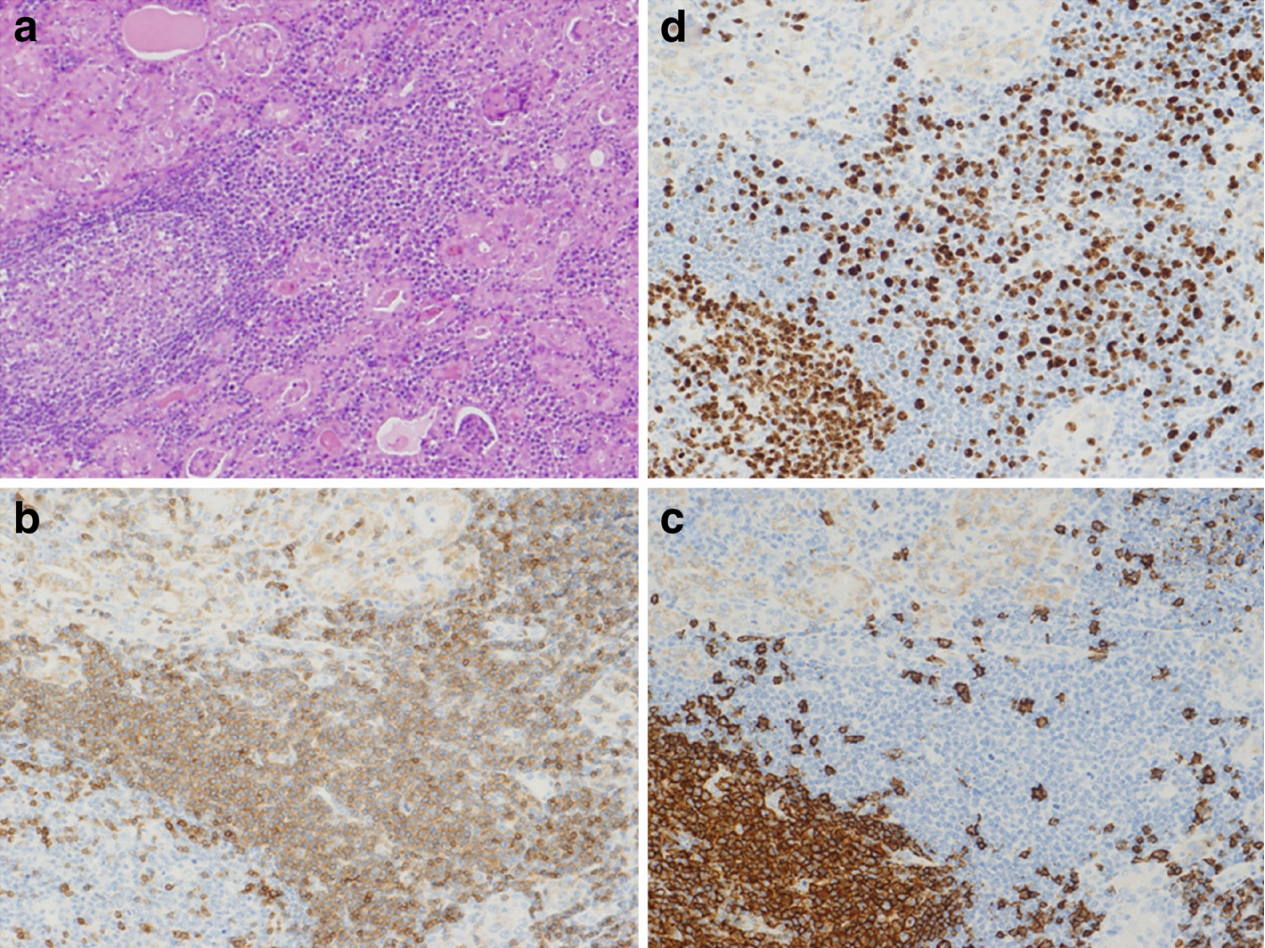
**Microscopic findings and immunohistochemical staining.** (**a**) Low-power view of histological examinations revealed massive infiltration of small monotonous lymphocytes, which were difficult to distinguish tumor cells from reactive lymphocytes in Hashimoto’s thyroiditis. (Hematoxylin and eosin staining, X100). (**b**) Immunohistochemical staining showed that tumor cells had T-cell markers for CD3, (X400). (**c**) Immunohistochemical staining by CD20 showed infiltrated lymphoid cells had B-cell markers, (X400). (d) MIB staining. MIB 1 index was as high as 60% in high-power fields, (X400).

In the medullary area, infiltrated lymphoid cells were relatively uniform, small-to-medium sized, round cells with a high nuclear to cytoplasmic (N/C) ratio. These cells had round nuclei with increased coarse chromatin and small nucleoli. Mitotic figures were often seen as high as 60% in high-power fields (Figure 3). These findings suggested small lymphocytic lymphoma. The atrophic thyroid epithelia showed enlarged and eosinophilic granular cytoplasm with large nuclei, so-called Hürthle cell metaplasia. Around these Hürthle cells there were many plasma cells, lymph follicles with germinal centers and a few eosinophils (Figure 3). These findings suggested complication of Hashimoto’s thyroiditis.

**Figure 4:**
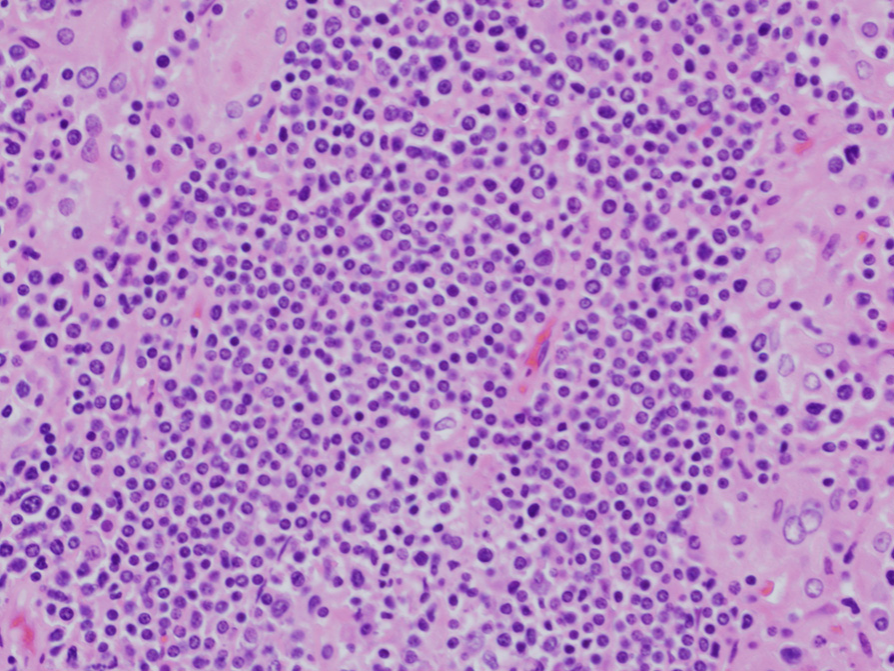
**High power magnification.** (Hematoxylin and eosin staining, X400). With the high-power view, tumor cells had a high nuclear to cytoplasmic (N/C) ratio, coarse nuclear chromatin with prominent nucleoli.

Immunohistochemical examinations were performed on formalin-fixed, paraffin-embedded specimens, using an autostaining system according to the manufacturer’s protocol. From these results, diffusely infiltrated lymphocytes were positive for T-cell markers (CD3 and CD45RO) and positive for B-cell markers (CD20). However, infiltrated atypical T cells showed CD3 dominancy (Figures 3 and 4). In contrast, lymphoid cells of the peripheral area with lymph follicles showed an admixture of T and B cells, mimicking lymph follicles.

### Flow cytometry

Surface marker analysis of the freshly resected tumor showed T-cell dominancy (CD2+, CD3+, CD4+, CD5+,CD45+). There was also a small population of B-cell lineage, with no dissociation of surface membranous immunoglobulin light chain kappa or lambda. These data suggest the polyclonal nature of infiltrated B lymphocytes associated with Hashimoto’s thyroiditis.

### Molecular analysis

**Figure 5:**
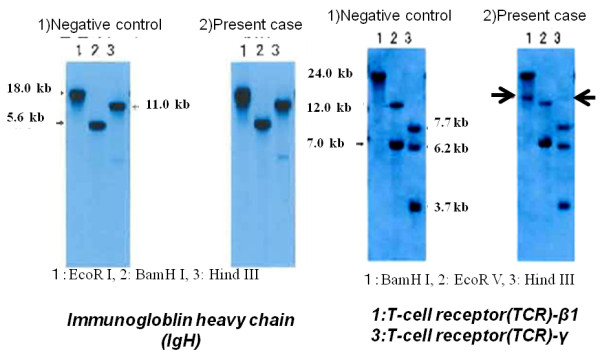
**Southern blot analysis (rearrangement).** (**a**) Rearrangement of the immunogloblin heavy chain (IgH) were negative, and (**b**) rearrangements of T-cell receptor (TCR)- β1 (lane 1) and TCR-γ (lane 2) were found in the present case. Rearrangement of TCR- δ was negative in the present case. M, molecular weight marker; 1, normal control (human placental DNA); 2, present case. 1, BamH I; 2, EcoR V; 3, Hind III.

Polymerase chain reactions (PCR) for detection of rearrangement of immunoglobulin heavy chain (IgH) hypervariable region and T-cell receptor (TCR) were performed using fresh specimens as described previously [2]. PCR showed a polyclonal pattern for both IgH and TCR. Southern blot analysis produced a rearrangement band of *TCR-*γ by *Hin*d III digestion and *TCR-*β by BamH I digestion (Figure 5). There was no rearrangement of the *TCR-*δ chain or IgH.

## Discussion

We diagnosed peripheral T-cell lymphoma of the thyroid gland associated with Hashimoto’s thyroiditis using molecular analysis. No other organ involvement of lymphoma was demonstrated, including the lymph nodes, the mediastinum and the bone marrow. Therefore, we considered this case to be a rare primary T-cell lymphoma of the thyroid gland, clinical stage I E-A with bulky mass.

However, malignant lymphoma is a minor component of thyroid tumors, accounting for only 2 to 5% of all thyroid malignancies. Malignant lymphoma of the thyroid gland is often associated with Hashimoto’s thyroiditis [1]. Most thyroid lymphomas are of the B-cell type, including maltoma and diffuse large B-cell lymphoma. T-cell lymphoma of the thyroid gland is extremely rare with only 15 cases reported in literature (Table 1) [3-15]. Most patients had a past history of Hashimoto’s thyroiditis, which affected female subjects more than male subjects. Most patients also had a history of rapid thyroid enlargement, sometimes accompanied with hoarseness or dysphasia. Open biopsy was the most common method of diagnosis; however, it is difficult to make a reliable diagnosis of thyroid malignant lymphoma. In the present case, a hemithyroidectomy was performed without a biopsy. Progression of malignant lymphoma in the thyroid was suspected from the clinical course, such as rapid growth and an enlarged thyroid tumor associated with Hashimoto’s thyroiditis.

**Table 1:** Lists of previously reported thyroid T-cell lymphoma

References*	**Age/gender**	**Presentation**	**Chronic thyroiditis**	**HTLV-1**	**Histology**	**Molecular Diagnosis**	**Diagnostic intervention**	**Therapy**	**prognosis**
[3] 1977	73/F		N/A	N/A				S + R	alive at 24 months
[4] 1990	79/M	Goiter	N/A	N/A	Diffuse small cleaved		open biopsy	C + R	alive at 4 years
[4] 1990	80/F	Goiter	(+)	N/A	Diffuse small cleaved		Hemithyroidectomy	S	DUC
[5] 1996	64/F	Thyroid mass, Hypothyroidism	(+)	(-)	CD2+, CD3+, CD5+, CD45RO+	TCR-β, TCR-γ rearrangement	Total thyroidectomy	S + C + R	alive at 9 months
[6] 1997	59/F	Goiter	(+)	(-)	CD3+, CD4-, CD8-, CD19-	TCR-δ rearrangement	Open biopsy	C + R	alive at 22 months
[7] 1999	65/M	Thyroid mass, Horseness, dyspasia,	(-)	N/A	CD45RO+, CD45-, vimentin+		Open biopsy	C + R	died at 11 months
[8] 1999	39/F	Goiter, fever, dysphonia	(+)	N/A	CD30+, CD45RO+, CD3-, CD20-, CD79a-, CD21-		Open biopsy	C + R	alive at 1 year
[9] 2000	63/F	Thyroid mass, Hoarseness, dyspnea	(+)	N/A	CD45RO+, CD43+		Total thyroidectomy	S + C + R	alive at 3 years
[10] 2001	72/M	Thyroid mass, pressure, difficulty swallowing	(+)	N/A	CD4+		Right hemithyroidectomy	C + R	alive at 12 months
[11] 2005	71/F	Thyroid swelling, horseness, goiter	(+)	(-)	CD3+, CD45RO+, CD4+	TCR-β, TCR-γ rearrangement	Total thyroidectomy	S	alive at 25 months
[12] 2005	86/F	Swelling of neck	(-)	(-)	CD3e+, CD5+, TIA-1+ (T-cell restricted antigen)	TCR-Cβ rearrangement	Left hemithyroidectomy	S	alive at 2 years
[13] 2007	34/M	N/A	(-)	(-)	CD3+, CD5+, CD7-, CD43+, CD45RO+, CD20-		N/A	S + C + R	died at 13 months
[14] 2007	61/M	Thyroid mass	(+)	(-)	CD3+, CD4+,	TCR-β rearrangement	Open biopsy	C	alive at 4 years
[14] 2007	68/M	Thyroid mass, dyspnea	(+)	(-)	CD3+, CD4+, TCR-ab+		Open biopsy	C	died at 5 months
[15] 2008	32/M	Swelling of neck, fatigue, shortness of breath	(-)	N/A	CD3+,	TCR-γ rearrangement(PCR)	Open biopsy	C	alive at 1 year
present	70/F	Neck swelling	(+)	(-)	CD3+, CD45RO+,	TCR-β, TCR-γ rearrangement	Total thyroidectomy	S	alive at 20 months

References*, Number in brackets indicate reference numbers and other numbers indicate published year; N/A, not available; C, chemotherapy; DFS, disease-free survival; DUC, died of unrelated cause; IgH, immunoglobulin heavy chain; PCR, polymerase chain reaction; R, radiation therapy; S, surgery; TCR, T-cell receptor.

Histological examinations revealed massive infiltration of small monotonous lymphocytes, which were difficult to distinguish from reactive lymphocytes in Hashimoto’s thyroiditis.

Immunohistochemical examination showed that these lymphocytes had T-cell markers (CD3 and CD45RO). However, the first pathological diagnosis could not accurately diagnose T-cell lymphomas. There are some reasons for the misdiagnosis in the current case. First, Hashimoto’s thyroiditis induced complicated massive infiltration of small lymphocytes, which were stained with B cell markers and T cell markers. It was difficult to distinguish tumor cells from reactive lymphocytes in Hashimoto’s thyroiditis. Second, T-cell lymphomas of the thyroid gland are very rare and cannot be easily referred to as an indicator. Third, present pathology has developed into various subdivisions and many pathologists have not always specialized in detecting malignant lymphoma disease.

On the absence of a reliable immunohistochemical marker of clonality of T-lymphocytes, genetic study is recommended as the most useful method to detect the presence of a dominant T-cell clone in a lymphocytic infiltrate [16,17]. After the 1990s, developments of molecular diagnosis, primary T-cell lymphomas of the thyroid gland were detected accurately, and slightly more cases were reported than before the 1980s. In the present case, PCR could not detect the monoclonality of the TCR; however, monoclonality of the proliferated lymphoid cells was seen on Southern blot analysis.

T-cell lymphomas are generally considered to have worse prognoses than B-cell lymphomas. However, with the exception of three, all reported patients with T-cell lymphomas did not die from the disease.

Treatment of thyroid T-cell lymphoma has focused on a combination of chemoradiotherapy and surgery, but no consensus has been reached. In the present case, only surgical resection was performed without chemotherapy or irradiation. This was because histology of the tumor showed that it was a low-grade lymphoma without extra-capsule spread. In addition, pathologically we found lymph node metastasis. Furthermore, the patient was a little old for chemotherapy and T cell lymphoma is generally resistant to chemotherapy or irradiation. Based on the literature available, we considered that adjuvant chemoradiotherapy could not contribute to a better prognosis than surgery alone (Table 1).

However, further investigations are needed to clarify this matter.

## Conclusions

Here we emphasized that a very rare case of peripheral T-cell lymphoma of thyroid gland with Hashimoto’s thyroiditis was effectively diagnosed by the gene rearrangement procedure. Particularly, patients with rapid thyroid enlargement accompanied with Hashimoto’s thyroiditis should also be examined by the gene rearrangement procedure.

## Consent

Written informed consent was obtained from the patient for publication of this case report and accompanying images. A copy of the written consent is available for review by the Editor-in-Chief of this journal.

## Abbreviations

CT, Computed tomography; FDG-PET, 18 F-Fluorodeoxy glucose-Positron Emission Tomography; TCR, T-cell receptor; PCR, Polymerase chain reactions; IgH, Immunoglobulin heavy chain.

## Competing interests

The authors declare that they have no competing interests.

## Authors’ contributions

JY and SI prepared and edited this manuscript. SO, ES, NK and MF contributed to the collection of data. MH performed the statistical analysis. JY and KI gave final approval for this version of the manuscript. All authors read and approved the final manuscript.

## References

[B1] Jaffe E Harris NL Stein H Vardiman L (Eds) World Health Organization Classification of Tumours. Pathology and Genetics of Tumours of Haematopoietic and Lymphoid Tissues Lyon, France: IARC Press; 2001

[B2] Diss TC Watts M Pan LX Burke M Linch D Isaacson PG The polymerase chain reaction in the demonstration of monoclonality in T cell lymphomas J Clin Pathol 1995 48 1045 1050 10.1136/jcp.48.11.1045 8543629 PMC503012

[B3] Dunbar JA Lyall MH MacGillivray JB Potts RC T-cell lymphoma of the thyroid BMJ 1977 2 679 302727 10.1136/bmj.2.6088.679 PMC1631914

[B4] Mizukami Y Michigishi T Nonomura A Nakamura S Hashimoto T Katsuda S Otake S Matsubara F Primary lymphoma of the thyroid: a clinical, histological and immunohistochemical study of 20 cases Histopathology 1990 17 201 209 10.1111/j.1365-2559.1990.tb00708.x 2242848

[B5] Abdul-Rahman ZH Gogas HJ Tooze JA Anderson B Mansi J Sacks NP Finlayson CJ T-cell lymphoma in Hashimoto’s thyroiditis Histopathology 1996 29 455 459 10.1046/j.1365-2559.1996.d01-515.x 8951491

[B6] Yamaguchi M Ohno T Kita K (gamma)/(delta) T-cell lymphoma of the thyroid gland N Engl J Med 1997 336 1391 1392 10.1056/NEJM199705083361915 9139227

[B7] Coltrera MD Primary T-cell lymphoma of the thyroid Head Neck 1999 21 160 163 10.1002/(SICI)1097-0347(199903)21:2<160::AID-HED10>3.0.CO;2-F 10091985

[B8] Forconi F Bocchia M Marconcini S Bigazzi C Milani M Fraternali-Orcioni G Lauria F CD30 positive (nonanaplastic) peripheral T-cell lymphoma of the thyroid gland Haematologica 1999 84 946 948 10509044

[B9] Haciyanli M Erkan N Yorukoglu K Sagol O Harmancioglu O Primary non-Hodgkin’s T-cell lymphoma of the thyroid gland complicating Hashimoto’s thyroiditis: case report Thyroid 2000 10 717 720 10.1089/10507250050137824 11014319

[B10] Raftopoulos I Vanuno D Kouraklis G Two unusual sites of colon cancer metastases and a rare thyroid lymphoma. Case 3 Primary T-cell lymphoma of the thyroid arising in a background of Hashimoto’s thyroiditis J Clin Oncol 2001 19 3576 3580 11481367 10.1200/JCO.2001.19.15.3576

[B11] Motoi N Ozawa Y Malignant T-cell lymphoma of the thyroid gland associated with Hashimoto’s thyroiditis Pathol Int 2005 55 425 430 10.1111/j.1440-1827.2005.01848.x 15982218

[B12] Okamoto A Namura K Uchiyama H Kajita Y Inaba T Nakamura S Shimazake C Cytotoxic T-cell non-Hodgkin’s lymphoma of the thyroid gland Am J Hematol 2005 80 77 78 10.1002/ajh.20393 16138337

[B13] Colović M Matić S Kryeziu E Tomin D Colović N Atkinson HD Outcomes of primary thyroid non-Hodgkin’s Lymphoma: a series of nine consecutive cases Med Oncol 2007 24 203 208 10.1007/BF02698041 17848745

[B14] Koida S Tsukasaki K Tsuchiya T Harasawa H Fukushima T Yamada Y Ohshima K Kamihira S Kikuchi M Tomonaga M Primary T-cell lymphoma of the thyroid gland with chemokine receptors of Th1 phenotype complicating autoimmune thyroiditis Haematologica 2007 92 37 40 10.3324/haematol.10351 17405755

[B15] Yang H Li J Shen T Primary T-cell lymphoma of the thyroid: case report and review of the literature Med Oncol 2008 25 462 466 10.1007/s12032-008-9059-x 18363111

[B16] Singer JA Primary lymphoma of the thyroid Am Surg 1998 64 334 337 9544144

[B17] Fodinger M Buchmayer H Schwarzinger I Simonitsch I Winkler K Jager U Knobler R Mannhalter C Multiplex PCR for rapid detection of T-cell receptor-gamma chain gene rearrangements in patients with lymphoproliferative diseases Br J Haematol 1996 94 136 139 10.1046/j.1365-2141.1996.6372268.x 8757524

